# Influence of incentive mechanism and fit degree on user’s environmental behavior—Taking Alipay “Ant Forest” in China as an example

**DOI:** 10.3389/fpsyg.2022.1033553

**Published:** 2022-12-20

**Authors:** Na Xiong, Ping Ren, Bingteng Sun, Surong He, Linli Jiang, Haitao Cui

**Affiliations:** ^1^School of Management, Guangxi Minzu University, Nanning, Guangxi, China; ^2^School of Economics, Guangxi Minzu University, Nanning, Guangxi, China; ^3^School of Economics and Management, Wuyi University, Jiangmen, Guangdong, China

**Keywords:** Ant Forest, game incentive, environmental behavior, stimulus-organism-response model, self-determination model

## Abstract

How to use game elements to motivate users and influence their behavior has become a new research trend, which is vital for enhancing the willingness of potential platform users to participate in environmental protection. This paper aims to analyze the influence of incentive mechanism and fit degree on user’s environmental behavior based on the stimulus-organism-response theory and self-determination theory. The questionnaire data of 500 users was collected and the impact of incentives on user’s environmental behavior was analyzed by structural equation modeling. The results show that economic, value, and social incentives have a significant impact on user’s environmental behavior. Besides, the value and social incentives of “Ant Forest” game platform positively influence user fit (conscious participation, enthusiasm, and platform interaction), but the impact of economic incentive on platform interaction is not statistically significant. From the perspective of user fit, “Ant Forest” game platform can positively promote users to adopt environmental behavior, because it explores users’ needs from their perspective to give full play to the role of game incentives on users' environmental behavior. Additionally, this research provides the practical implications for managers exploring the effects of co-creation processes in developing countries and regions.

## Introduction

With the increasing maturity of internet plus technologies, it is rapidly integrated into people daily life, and various games occupy a number of people’s fragmented time. Recently, Newzoo, as a market research firm, has found that the global mobile game market would reach $90.7 billion in an analysis of the 2021 global game market data, compared with China accounting for 45% of the overseas mobile game market. Reviewing the phylogeny of games, the first video game is 7 years longer than the internet in the world. As the spiritual level that people are pursuing is increasing day by day, their pursuit of games is not only in the sensory experience. Therefore, the platform begins to try to integrate various game elements into new things and hotspots, which not only encourages users to actively participate, but also can enhance the stickiness and retention rate of users, thereby improving game marketing communication performance. Nowadays, how to use game elements to motivate users and influence their behavior has become a new research trend. Among them, “Ant Forest” adopts this marketing model.

“Ant Forest” is an environmentally friendly game product launched by China Alipay APP, which is mainly used to detect the reduction of carbon emissions in people’s daily life. By metering daily low-carbon behavior into green energy (virtual energy), when a certain amount of green energy is accumulated, the nonprofit will plant a real tree. Moreover, it is different from environmental protection game software abroad, such as Pollushot, whose purpose is to arise users’ satisfaction for use, thereby enhancing the willingness to use (Zhang et al., 2020). As a consequence, it is significant to determine what factors stimulate users’ eco-friendly behavior. Games are known to stimulate the potential for multiple beneficial outcomes, including fostering intrinsic motivation (Xu et al., 2021), cognitive (Berg, 2021), and emotional benefits (Kowal et al., 2021). Suits (1967) points out that games are activities using certain rules and specific means to engage. Gamification focuses on transferring game design elements into a non-game context (Dichev and Dicheva, 2022). Hence, Seaborn and Fels (2015) indicate that gamification must obey a goal, namely, to have an impact on users. Rapp (2017) has found that the design of human-computer interaction can effectively improve the users’ participation, thereby enhancing the personal value of users.

Relevant scholars in the field of environmental psychology and environmental management have found that the impact of public environmental behavior can be divided into intrinsic motivations and extrinsic intervention. The intrinsic motivations include problem awareness, moral values, etc. (Brekke et al., 2003; Homburg and Stolberg, 2006; Turaga et al., 2010). On the contrary, extrinsic intervention mainly relies on relevant policy measures to change public behavior, including publicity and education, normative system, etc. (Schultz, 1999; Ma et al., 2021). The core of incentive is intrinsic motivation (Rheinberg and Engeser, 2018), the condition of incentive is external intervention (Frey and Jegen, 2001), and the purpose of incentive is behavior (Young, 1936). Therefore, incentives will affect the public environmental behavior. Some scholars have also explored the influence factors of public environmental behavior by introducing environmental protection attitude and environmental protection intention. The study has found that two variables have a positive and significant influence relationship (Khaleeli and Jawabri, 2021). Based on this, the game incentives are used to build a good interactive experience and internal connection between users and their environmental behavior.

Since the Brundtland Report (1987) was released, sustainable development has become a hot topic of our time and is of great interest to society, governments, and many international organizations (Sauvé et al., 2016; Nousheen et al., 2020). Reviewing the early literature, the environmental problem is not only a simple problem, but also a comprehensive problem of society, management and environment. The main solutions considered educational awareness, social influence, encouragement, behavioral insight and incentive, which have been used in experimental studies (Grilli and Curtis, 2021). Among them, social influence is the impact of other people on individuals (Latané, 1981). Some of the subsequent challenges faced by society include lack of political will, inadequate funding, and insufficient behavior to engage in environmental activities (Oruonye et al., 2021). Fortunately, “Ant Forest” breaks down these difficulties and enables people to engage in environmental behavior. Although the cost of users’ time spent per game play is immediate, the benefits of their active participation in environmental behavior during the game play are long-term, thus contributing to environmental sustainability. In other words, the public plays an important role in ecological governance as a major actor in environmental protection (Lihua et al., 2020). So public support will build a virtuous cycle of society, management, and environment.

From the above-mentioned literature, the existing literature tends to focus on the factors influencing public environmental behavior, including environmental awareness and environmental attitude. A variety of beneficial potentials can be stimulated with the help of games, but game incentives are often introduced as external factors that influence users’ participation in environmental behavior. At present, as few studies have explored the psychological influence of game incentives on users’ environmental behavior, this study combines the stimulus-organism-response theory (SOR) and self-determination theory (SDT) to trace users’ psychological motivations and induce their interest in environmental behavior. The questionnaire data of 500 users was collected and the structural equation modeling was used to analyze the influence of various incentives and user fit on their environmental behavior, which provides a reference for subsequent exploring the factors influencing the public’s environmental behavior.

## Theoretical model and hypotheses

### Stimulus–organism–response and self–determination theory

Based on the literature review in the first part, it can be concluded that game incentives are a new type of performance to improve users’ environmental behavior. The stimulus-organism-response (SOR) theoretical model was first designed and applied to environmental psychology by Mehrabian and Russell (1974). This theory has been widely used in tourism, e-commerce, consumer buying behavior, etc. (Guo et al., 2021; Karim et al., 2021; Qiu et al., 2022). They state that the external environment stimulates the psychological, emotional, and cognitive changes in individuals, leading to a range of behavioral outcomes. In recent years, scholars have gradually studied this model in the context of cognition and participation in environmental behavior (Wei et al., 2020; Song et al., 2022). In exploring what factors drive residents’ energy-saving behavior, the SOR theoretical model is introduced to explore the mechanisms of environmental stimuli (S) and internal psychological states (O) on residents’ energy-saving behavior (R) (Ding et al., 2022). In exploring the influence of online platforms on green consumption behavior, combining the SOR model with online platforms as stimulus (S), environmental attitudes as organism (O), and green consumption ability as response (R), it is concluded that online platforms promote users’ green ability (Ma and Liu, 2022). Similarly, this study can use this approach to explore the influence of game incentives on users’ environmental behavior. That is, game incentive (S) and user adaptation (O) stimulate and lead to the user's response to adopt specific environmental behavior (R). The SOR theoretical model is more appropriate to the topic of this study, and it constructs a theoretical model of the relationship between game incentives, user fit, and environmental behavior in this research area.

The self-determination theory (SDT) is an empirically derived theory of human motivation and personality in social contexts that differentiates motivation in terms of being autonomous and controlled. Work leading to the theory began with experiments examining the effects of extrinsic rewards on intrinsic motivation (Deci and Ryan, 2012). At present, SDT is often widely used in consumer behavior theory, especially in the field of gamification, and is usually used to explore the reasons why users participate or make a decision in games (Tobon et al., 2020). The SDT holds that three types of motivation, namely intrinsic motivation, extrinsic motivation, and amotivation, and it can induce people to participate in an activity. Among them, intrinsic motivation refers to the fact that the thing itself can attract people’s interest, and these motivations do not come from outside (Ryan and Deci, 2000). Furthermore, owing to their entertainment and fun features, games can motivate people to continuously generate intrinsic behavioral motivations (Lazzaro, 2009). Since the incentive effect of the game is not changed by the external environment, the external reward will stimulate the changes in the users’ psychological state, thereby affecting the users’ behavioral intention. Therefore, this study can use the SDT model to analyze the game incentive mechanism in detail and analyze whether the reward mechanism in “Ant Forest” satisfies users and attracts their active participation, delivering brand information and content in the process.

### Incentive mechanism of “Ant Forest” game

Gamification is becoming more and more common as a means of motivating users to engage with the platform. During the game, users will focus on getting more rewards or their own location at the top of the honors received (Easley and Ghosh, 2016). Reviewing the literature, we find that incentives are mainly developed in economic incentive which can stimulate active participation of users, leading to more proactive behavior (Arriagada et al., 2022). Common game elements are points, badges, leaderboards, etc. (Saleem et al., 2021). Users gain badges and rankings while their own value is increased. This incentive is identified as a common gamification mechanism (Tobon et al., 2020). Phillips (1967) stated that social engagement increases self-happiness, and adding social incentive increases the willingness of users to adopt behavior. Based on the above analysis, the following hypotheses are proposed.

*Hypothesis 1a*: Economic incentive positively affects the environmental behavior of “Ant Forest” users.

*Hypothesis 1b*: Value incentive positively affects the environmental behavior of “Ant Forest” users.

*Hypothesis 1c*: Social incentive positively affects the environmental behavior of “Ant Forest” users.

### User fit

At present, scholar concept of user fit is presented through multiple dimensions. According to the relevant literature review, most scholars use the three-dimensional division method. Hollebeek et al. (2014) and Jaakkola and Alexander (2014) also divide it into three levels of cognition, emotion, and behavior in the research of user fit. Different from most scholar research, Vivek et al. (2012) defines the three dimensions of user engagement as conscious participation, enthusiasm, and social interaction. The fundamental significance is to make the three dimensions of user fit more realistic. In other words, user conscious participation corresponds to cognition, enthusiasm corresponds to emotion, and social interaction corresponds to behavior. Through the above literature research, it finds that the dimensions of user engagement are inseparable from cognition, emotion and behavior, but the refined analysis of user engagement is based on the different scenarios between users and various companies. As a consequence, this paper chooses Vivek division method of user fit, and divides the user fit of Alipay “Ant Forest” game platform into three categories of conscious participation, enthusiasm, and platform interaction. Based on the above analysis, the following hypotheses are proposed.

*Hypothesis 2a*: Economic incentive has a positive impact on users’ conscious participation.

*Hypothesis 2b*: Economic incentive has a positive impact on users’ enthusiasm.

*Hypothesis 2c*: Economic incentive has a positive impact on users’ platform interaction.

*Hypothesis 3a*: Value incentive has a positive impact on users’ conscious participation.

*Hypothesis 3b*: Value incentive has a positive impact on users’ enthusiasm.

*Hypothesis 3c*: Value incentive has a positive impact on users’ platform interaction.

*Hypothesis 4a*: Social incentive has a positive impact on users’ conscious participation.

*Hypothesis 4b*: Social incentive has a positive impact on users’ enthusiasm.

*Hypothesis 4c*: Social incentive has a positive impact on users’ platform interaction.

### User environmental behavior

Early scholars identified user fit as a motivation for adopting behavior and argued that successful user fit enhances users’ willingness to use (Lin, 2012). Linn et al. (1994) showed that environmentally conscious people will actively participate in environmentally friendly behavior to reduce waste emissions. Bissing-Olson et al. (2013) studied the green behavior of corporate employees and found that highly positive attitudes and enthusiasms toward the environment directly influence employees’ environmental behavior. Zhang et al. (2020) found that platform interaction enhances user satisfaction, which leads to positive behavior. Therefore, based on the above literature, three dimensions are proposed to influence the adoption of environmental intention.

*Hypothesis 5a*: Conscious participation has a positive impact on the environmental behavior of users using “Ant Forest.”

*Hypothesis 5b*: Enthusiasm has a positive impact on the environmental behavior of users using “Ant Forest.”

*Hypothesis 5c*: Platform interaction has a positive impact on the environmental behavior of users using “Ant Forest.”

**Figure 1:**
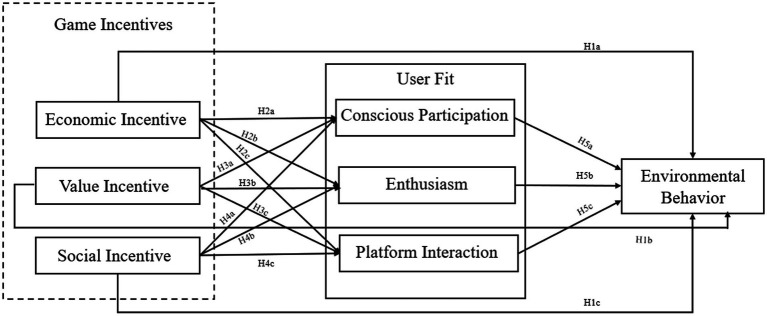
Research model diagram.

To sum up, this paper proposes a theoretical model of the influence of the “Ant Forest” Game Incentives Mechanism on the willingness of potential users to adopt environmental behavior, as shown in Figure 1.

## Research design

### Sample and data collection

The survey sample sources were quantitatively collected online *via* electronic questionnaire. The main advantage lied in the wide range of survey objects, and the online survey was mainly entrusted to a third-party “questionnaire network”. Based on our research questions, the objects of this survey should be the population who know the game of “Ant Forest”. A total of 550 online questionnaires were distributed. Among them, a total of 500 online questionnaires were collected, 50 invalid questionnaires were excluded. The effective recovery rate was 90.91%, meeting the large sample requirements proposed by structural equation modeling. The main reasons for the invalid questionnaire: (1) The respondents did not understand the game strategy of “Ant Forest” in Alipay. (2) There were obvious logical problems in the answers to the questionnaire. From the statistical characteristics of the respondents, it can be implied that the rate of male and female is basically the same. The age group in this study is between 18 and 30 years old. A similar distribution is shown in the national statistics. It also shows that the target users of game incentives are more popular among young people. The number of samples with a bachelor degree or above reaches 410, accounting for 80%. In conclusion, the sample selected for this study is suitable, and Table 1 below provides descriptive statistics analysis on the collected data.

**Table 1:** Demographic characteristics of the survey sample.

Users’ characteristic		Level	Percentage
Gender	Male	230	46.0
	Female	270	54.0
Age	Under 18	22	4.4
	18–30	437	87.4
	31–40	30	6.0
	41–50	6	1.2
	Over 51	5	1.0
Education level	Under College	90	18.0
	Bachelor	278	55.6
	Master	122	22.4
	Doctor	10	2.0

### Variables selection

In order to ensure the reliability and validity of the scale, before the formal questionnaire was opened, offline interviews were conducted with potential users and a small sample pre-test was taken. Then minor modifications to the scale were made to form a formal scale. The seven latent variables of this study are designed on the basis of relevant literature, with a total of 21 items. Among them, the game incentive methods of “Ant Forest” are divided into three levels of economic incentive, value incentive, and social incentive. When users participate in the “Ant Forest” gamification application, the platform will directly distribute forest props to users, get beautiful costumes for users, and increase the number of good fortune through environmental protection in order to divide the cash, which reflects economic incentive. The frequency of users' participation in the "Ant Forest" will improve the level, score and fulfillment of users in this application, which is the value incentive for users. The "Ant Forest" application also sets up some social rewards, for example, the task of planting green trees between users and completing tasks through mutual cooperation, which is a social incentive for users. This paper chooses Vivek division method of user fit, and divides the user fit of Alipay “Ant Forest” game platform into three categories of conscious participation, enthusiasm, and platform interaction to influence potential user perception of environmental behavior. By making appropriate adjustments on the basis of the mature scales of other scholars, a formal questionnaire was eventually designed. In order to facilitate subsequent research and analysis, as shown in Table 2, the variables in the following scales are renamed as follows:

**Table 2:** Scales of the relevant variables.

Variable	No.	Item
Economic incentive (EI)	EI1	In “Ant Forest,” in order to obtain forest props, I am motivated to take different environmental behavior.
	EI2	In “Ant Forest,” in order to get different costumes, I am motivated to adopt different environmental behavior.
	EI3	In “Ant Forest,” in order to increase the number of good fortune, I am motivated to adopt different environmental behavior.
Value incentive (VI)	VI1	I feel honored when I “plant” a tree.
	VI2	Every time I enter a new stage level, it motivates me to adopt different environmental behavior.
	VI3	Whenever I am at the top of the weekly rankings, I think the time and experience on “Ant Forest” is worth it.
Social incentive (SI)	SI1	Using “stealing” energy from friends in the “Ant Forest” will create our own circle of friends.
	SI2	The cooperative “tree planting” mission of “Ant Forest” prompts me to adopt different environmental behavior to obtain more energy.
	SI3	In the “Ant Forest,” watering my friends prompts me to adopt different environmental behavior to get more energy.
Conscious Participation (C)	C1	I will take the initiative to pay attention to which environmental behavior will get more incentives for environmental protection in games.
	C2	I would like to know more about the content in “Ant Forest” to improve the game’s environmental incentive.
	C3	The game environmental incentive in “Ant Forest” can attract my attention.
Enthusiasm (E)	E1	I am very interested in the game environmental incentive of “Ant Forest.”
	E2	I like the environmental protection games provided by the platform of “Ant Forest.”
	E3	Setting up a watering game in “Ant Forest” makes a difference in my social life.
Platform interaction (I)	I1	I like to work with others to plant trees so that I can increase the number of environmentally friendly behavior.
	I2	When I watering my friends, it is more about getting more energy by increasing the number of eco-friendly behavior.
	I3	I find it more interesting when the people around me also complete the task of “planting trees.”
Environmental behavior (EB)	EB1	Since playing “Ant Forest” game, my routine has changed, I will get up in the morning to collect energy.
	EB2	In my daily life, I prefer a lifestyle that gets more green energy from “Ant Forest.”
	EB3	I will use “Ant Forest” frequently in the future.

## Model analysis and hypothesis testing

### Reliability and validity testing

AMOS.26 software is used in this study to verify the construct validity of the valid questionnaires. From the test results, it finds that the test value of Cronbach’α is 0.856, indicating that the questionnaire has good reliability. The Cronbach’α coefficients are all above 0.7, indicating that the scale has good internal consistency, and the model used in the research survey has high reliability.

As shown in Table 3, this paper uses factor loading and average variance extracted (Average Variance Extracted, AVE) to test the convergent validity of the model. It can be seen that the KMO value of each latent variable is above 0.6, and the Bartlett sphericity test results are all significant at the level of 0.000. Therefore, it indicates that the scale in the questionnaire is suitable for factor analysis, and further indicates that the scale and model of questionnaire have high reliability in this study. Through the confirmatory factor analysis of measured variables with the help of AMOS.26 software, the results show that the standardized factor loading value of each latent variable is mostly above 0.6, and the average variance extraction (AVE) is greater than 0.6. Consequently, it indicates that the scale has good internal consistency and convergent validity. Based on the above information, the model has high reliability and good convergent validity, and the validity and quality of each latent variable is excellent.

**Table 3:** Results of the confirmatory factor analysis.

Latent variable	Measure variable	Normalized factor loading	Cronbach’s α	AVE	KMO
Economic Incentive (EI)	EI1	0.833	0.872	0.711	0.742
	EI2	0.842			
	EI3	0.854			
Value Incentive (VI)	VI1	0.868	0.865	0.709	0.728
	VI2	0.814			
	VI3	0.824			
Social Incentive (SI)	SI1	0.847	0.898	0.734	0.740
	SI2	0.857			
	SI3	0.867			
Conscious Participation (C)	C1	0.814	0.797	0.607	0.685
	C2	0.811			
	C3	0.708			
Enthusiasm (E)	E1	0.839	0.867	0.687	0.718
	E2	0.848			
	E3	0.799			
Platform Interaction (I)	I1	0.883	0.910	0.801	0.748
	I2	0.906			
	I3	0.895			
Environmental Behavior (EB)	EB1	0.825	0.806	0.611	0.659
	EB2	0.838			
	EB3	0.670			

This paper uses the Pearson method to test the discriminant validity of each latent variable. Through correlation analysis, it finds that the correlation coefficient between each variable and other variables are less than the square root of the Average Variance Extracted (AVE). It shows that the questionnaire has good discriminant validity. At the same time, the variance inflation factor (VIF) of each variable is less than 10, indicating that there is no multicollinearity problem between the variables.

### Model fit test

The results of reliability, validity, and confirmatory factor tests show that this study is suitable for analysis using structural equation model. Using the AMOS.26 software to test the fit of the initial model, the results also show that the latent variables are sufficiently strong to interpret the explicit variable. The overall fit of the measurement model is up to standard, and the CMIN/DF is 2.453, between 1 and 3. The RMR value is 0.061, indicating that the model fits well with the actual sample data. In addition, the values of GIF, NFI, and RFI are 0.928, 0.934 and 0.919, respectively. At the same time, the values of IFI, TLI and CFI are all greater than 0.9 and reach a good level, which also shows that the fitting effect is good and has high effectiveness.

### Model parameter estimation

**Figure 2:**
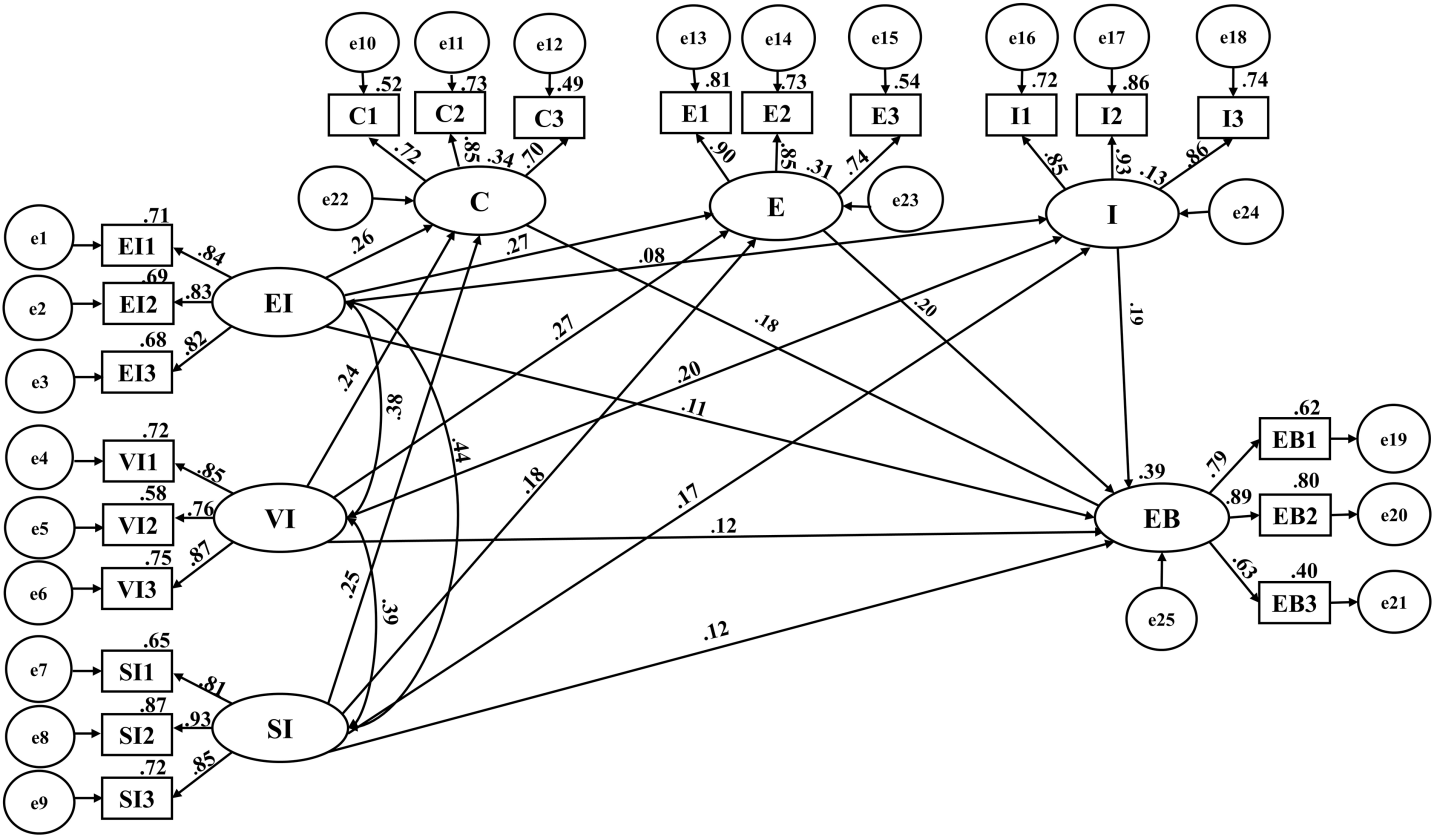
Estimation results of model path coefficients.

With the help of AMOS.26 software, structural equation model analysis was carried out to analyze the influence of “Ant Forest” game incentive mechanism on users’ environmental behavior. The specific analysis results are shown in Figure 2, and the model parameter estimation results are shown in Table 4.

1. Economic, value, and social incentive all have a significant impact on users’ environmental behavior, which supports the assumptions above, and the path coefficients are 0.102, 0.100, and 0.089 respectively. From the perspective of the impact of the three on users’ environmental behavior, economic incentive is greater than value and social incentive, and social incentive has the least impact, which fully reflects the importance of economic incentive to potential users.
2. In the process of this research, the incentive methods of games are divided into three dimensions, namely economic, value, and social incentive. As a consequence, exploring the influence of “Ant Forest” game incentive and user fit should be carried out from three dimensions below. First of all, the path coefficient of economic incentive to users’ conscious participation is 0.213, the path coefficient of economic incentive to enthusiasm is 0.350, and the path coefficient of economic incentive to platform interaction is 0.093. We can see that the impact of economic incentive on platform interaction is not statistically significant. It is likely that this incentive method fails to make users feel the purpose of the platform. In the actual investigation, it is also found that the game environmental incentive provided by “Ant Forest” could not fully attract users’ attention. As a consequence, it is assumed that H2a, and H2b are all established. That is, economic incentive has a positive impact on user engagement. Secondly, the path coefficient of value incentive to users’ conscious participation is 0.171, the path coefficient of value incentive to enthusiasm is 0.306, and the path coefficient of value incentive to platform interaction is 0.206. As a result, assuming that H3a–H3c is all established. That is, value incentive has a positive impact on user engagement. Finally, the path coefficient of social incentive to users’ conscious participation is 0.172, the path coefficient of social incentive to enthusiasm is 0.192, and the path coefficient of social incentive to platform interaction is 0.164. Hence, it is assumed that H4a–H4c is all established. That is, social incentive has a positive impact on user engagement.
3. Users’ conscious participation, enthusiasm, and platform interaction have a significant and prominent impact on users’ environmental behavior, which can be concluded from the path analysis. The path coefficients for conscious engagement, enthusiasm, and platform interaction are 0.206, 0.138, and 0.150, respectively. Among them, the significance of platform interaction and enthusiasm are higher, reaching 0.000, followed by conscious participation. This also reflects from the side that “Ant Forest” can actively prompt users to adopt environmental behavior. As a consequence, if users can be more consciously involved, it will be more conducive to expand the number of users.

**Table 4:** Estimation results of structural equation model.

Hypothesis	Path coefficient
*H1a*: Economic Incentive→Environmental Behavior	0.102^*^
*H1b*: Value Incentive→Environmental Behavior	0.100^*^
*H1c*: Social Incentive→Environmental Behavior	0.089^*^
*H2a*: Economic Incentive→Conscious Participation	0.213^***^
*H2b*: Economic Incentive→Enthusiasm	0.350^***^
*H2c*: Economic Incentive→Platform Interaction	0.093
*H3a*: Value Incentive→Conscious Participation	0.171^***^
*H3b*: Value Incentive→Enthusiasm	0.306^***^
*H3c*: Value Incentive→Platform Interaction	0.206^***^
*H4a*: Social Incentive→Conscious Participation	0.172^***^
*H4b*: Social Incentive→Enthusiasm	0.192^***^
*H4c*: Social Incentive→Platform Interaction	0.164^**^
*H5a*: Conscious Participation→Environmental Behavior	0.206^**^
*H5b*: Enthusiasm→Environmental Behavior	0.138^***^
*H5c*: Platform Interaction→Environmental Behavior	0.150^***^

*, ** and *** represent significant at 10%, 5% and 1%, respectively.

### Mult-group analysis

In order to further test the moderating effect of users’ demographic characteristics on users’ environmental behavior, this paper divides the overall sample into different subgroups for analysis according to the gender, age, and educational level of potential users. Because the data are relatively scattered, the age group is divided into the group under 30 and over 31. The educational level is divided into the low-level group (undergraduate and below) and the high-level group (postgraduate and above). The results are shown in Table 5. As a whole, it can be concluded that there are certain differences in significance and impact size between groups and between the original total samples, but the positive and negative relationship of the standardized path coefficients are basically the same as that of the total samples, as follows:

1. Gender group: From the impact path of economic incentive on users’ environmental behavior, it can be seen that the female group has a significant impact. On the contrary, the male group has no important impact. The same is true in the impact of conscious participation on environmental behavior. From the impact path of value incentive on enthusiasm, we can be seen that both have an impact, but the impact on the male is more significant. Because the female is not sensitive to value incentive. Compared with the male, beautiful clothes attract more female and make them more willing to take more environmental behavior to harvest more energy.
2. Age group: in the influence path of value and social incentive on user fit, the moderating effect of group aged 31 and above is not significant, while the impact of user fit on users’ environmental behavior is significant. On the contrary, except that the impact of economic incentive on platform interaction is not significant, all others have a significant impact on users’ environmental behavior. This shows that the “Ant Forest” is more used by young people. Compared with the complexity of game strategy, young people are more willing to explore, while older users feel distressed. Therefore, there are some differences.
3. Education level group: in the influence path of economic incentive on users’ environmental behavior, the low-level group is not significant. On the contrary, the high-level group is significant. In the impact path of value and social incentive on users’ environmental behavior, the high-level is not significant. The impact of value incentive on user fit is not different in the education level, and it is significant which is completely consistent with the previous research results. Other incentive measures are significantly different among different level. Table 5 shows that the low-level group is more affected and the high-level group is relatively less affected. The reason might be that the high-level group has less leisure time. Therefore, their ability to obtain relevant game strategy information is generally lower than the low-level group. The results of this research also provide accurate positioning that users are the main movers of the “Ant forest” game.

**Table 5:** Estimation results of multi-group analysis.

Hypothesis	Gender		Age		Education level	
	Male	Female	Under 30	Over 31	Low-level	High-level
*H*1a	0.021	0.196^**^	0.131^*^	−0.236	0.071	0.179^*^
*H*1b	0.159^*^	0.072	0.164^**^	−0.241^*^	0.163^*^	0.093
*H*1c	0.077	0.144^*^	0.103^*^	0.297^*^	0.082	0.132
*H*2a	0.397^***^	0.158^*^	0.226^***^	0.584^**^	0.304^***^	0.136
*H*2b	0.351^***^	0.221^**^	0.222^***^	0.656^***^	0.367^***^	−0.002
*H*2c	0.130	0.045	0.057	0.232	0.157^*^	−0.096
*H*3a	0.192^*^	0.276^***^	0.255^***^	−0.036	0.222^***^	0.274^**^
*H*3b	0.300^***^	0.229^**^	0.294^***^	−0.031	0.288^***^	0.251^**^
*H*3c	0.226^***^	0.171^**^	0.197^***^	0.178	0.210^**^	0.207^*^
*H*4a	0.209^**^	0.266^***^	0.260^***^	0.281	0.304^***^	0.136
*H*4b	0.116	0.219^**^	0.203^***^	0.032	0.100^*^	0.357^***^
*H*4c	0.192^*^	0.159^*^	0.182^**^	0.118	0.096	0.342^**^
*H*5a	0.087	0.322^***^	0.158^**^	0.525^*^	0.275^***^	−0.042
*H*5b	0.242^**^	0.155^*^	0.186^**^	0.321^*^	0.174^**^	0.281^**^
*H*5c	0.244^***^	0.115^*^	0.174^***^	0.310^*^	0.171^**^	0.212^*^

*, ** and *** represent significant at 10%, 5% and 1%, respectively.

## Conclusion and discussion

### Conclusion

In this paper, we proposed a conceptual model of the influence of users’ environmental behavior and conducted an empirical test to investigate how to use game elements to motivate users and influence their behavior in “Ant Forest.” The empirical investigation was conducted through questionnaires, and the following conclusions were drawn. Firstly, by using SOR theory to construct a new model to stimulate users to adopt environmental behavior, it is found that the game incentivizes different groups of people to adopt environmental behavior. It also reflects that the incentive mechanism affects the public’s environmental behavior. Secondly, by referring to the three-dimensional subdivision method of Vivek et al. (2012), we divide the user fit into three dimensions, namely conscious participation, enthusiasm, and platform interaction, to explore their impact on user environmental behavior. Among them, most users prefer to use "Ant Forest" in the future, which reflects the positive interaction between the platform and users. Finally, by using SDT theory, the game incentives are subdivided. When exploring the impact of subdivided game incentives on user fit, this paper finds that most of the incentive will improve user fit. However, economic incentive do not have a significant impact when it comes to platform interaction.

### Discussion

The problems of resource shortage and environmental pollution damage have become a worldwide focus (Cai and Tang, 2021; Murphy et al., 2021). Furthermore, the environmental protection has always been the key topic of social concern. “Ant Forest” provides a way based on the information technology, which can enable people to engage in green behavior (Zhang et al., 2020). As a consequence, analyzing the game mechanism of “Ant Forest” is extremely significant for users to participate in environmental protection.

The SOR theory is used to analyze a model of human behavior (Jacoby, 2002). The existing researches illustrate that game elements stimulate user’s behavior (Yuanyuan and Liangmou, 2021). For instance, emotions or personal achievements of users will have a significant impact on environmental behavior in “Ant Forest” (Moore and Yang, 2020; Mi et al., 2021). However, few people have conducted correlative researches in game motivation. The game incentives are a type of game elements (O’Donovan et al., 2013), resulting that gamification incentives have a positive effect on user’s environmental behavior. In this paper, we divide incentives into the economic, value, and social incentive by SDT model, which can be transformed into emotional stimulation to motivate users to implement more environmental behavior. In addition, the gamification is to create a sense of game in a non-game environment so that the gamification can make users happy in the process of participation (Thom et al., 2012). Consequently, users will be more willing to use “Ant Forest.”

Different from the previous researches on the impact of game mechanism on users’ environmental behavior, this study collected data through questionnaire survey and evaluated it with the structural equation modeling to explore the incentive methods that users like, resulting that users can make more environmentally friendly behavior. The structural equation modeling is a method to estimate and test the causality model (Hox and Bechger, 1998), which is often used in theoretical research of social sciences (Babin and Svensson, 2012). On the one hand, the structural equation modeling is selected as the evaluation method for this study, which can simultaneously observe and analyze the explicit variable and potential variable, as well as can analyze whether each obvious variable is related to the potential variable. On the other hand, these variables of the research including attitude and behavior often contain errors, yet the analysis of structural equation modeling allows both independent and dependent variables to contain measurement errors. Moreover, the structural equation modeling can be used to evaluate and revise the model. As a result, it is reasonable to use structural equation method for evaluation.

Generally, this paper makes the following contributions. First, it aims to identify the classification of incentives, namely economic, value, and social incentive. Second, it introduces the user fit between game incentive and environmental behavior, and extends the three levels of cognition, emotion, and behavior to the conscious participation, enthusiasm and platform interaction. Third, by analyzing the impact of various incentives on users’ environmental behavior in the gamification incentive process, it breaks the single linear model thinking in previous gamification studies.

### Practical implications

Based on the above conclusions, the practical significance of managers’ exploring the effects of co-creation processes should be noticed. First, data sharing on the “Ant Forest” construction platform should be improved. In the era of vigorous development of green economy, these data are well prepared to drive economic development, and user preferences are better understood by managers through shared data in order to improve environmental protection efficiency. Second, government policies of digital transformation should be well targeted and faster in implementation. With the introduction of a series of digital transformation of environmental sustainability policies, China’s environmental protection industry has been effectively promoted, but its target is not strong, which to a certain extent will weaken its impact on environmental sustainability.

### Limitations and future research directions

In this paper, we provided an empirical verification and a new notion for the research of gamification incentives and environmental behavior, as well as the references for other stimulating behavior. However, it is worth noting that this paper is limited by the following deficiencies. Firstly, with respect to the distribution method of survey questionnaire, we used an online electronic questionnaire for quantitative collection. This method was forwarded by family members and friends in WeChat. Thus they might have many similar or consistent opinion when the users filled the questionnaire, which had a certain impact on the reliability and validity of our data. Secondly, we adopted the positive incentives including economic, value, and social incentive when subdividing the explanatory variables, but ignored the negative incentives consisting of the green recycling, green packaging, and other projects. Finally, the factors that affect users’ environmental behavior are not limited to the explanatory variables discussed in this paper, as well as other factors such as environmental behavior education (Ouariachi et al., 2020), personal satisfaction (Mi et al., 2021), etc.

In the future, the follow-up research should focus on the following aspects. First, it can highlight the potential role of environmental sustainability and digital transformation issues in the social sciences (Lim, 2016; Centobelli et al., 2018). In this context, using the digital transformation methods including artificial intelligence, big data analysis, internet of things, and other emerging technologies can solve more environmental problems and help ensure environmental sustainability (Feroz et al., 2021; Truong, 2022). Future research should focus on exploring how different types of digital transformation can affect environmental sustainability effectively in economic and social fields. Second, it can strengthen the intersection application of the green practices and digital technologies. The digital technology has been a boon in erstwhile management practices. Recently, the digital technologies have been embedded in the business (Gregori and Holzmann, 2020), supply chain (Song, 2019), and so on. In the future, the digital technology also can be embedded into green practice. In green practice, we should find the right fit and focus of digital technology to enable ecological environment protection, so as to promote the construction of green technology innovation system and strengthen application scenarios. This requires further multi-dimensional comprehensive research.

## Data availability statement

The original contributions presented in the study are included in the article/supplementary material, further inquiries can be directed to the corresponding author.

## Author contributions

All authors listed have made a substantial, direct, and intellectual contribution to the work and approved it for publication. NX conceived and designed this study. PR performed the empirical analysis of data and contributed significantly to manuscript preparation. BS revised the grammar and sentences of the manuscript. LJ contributed to revising the manuscript critically for important intellectual content. SH and HC put forward valuable idea and participated in writing the manuscript.

## Funding

Our research was approved and funded by the National Natural Science Foundation of China (Project number: 72003051) and the Ministry of Education of Humanities and Social Science Project of China (Project number: 19YJC790048).

## Conflict of interest

The authors declare that the research was conducted in the absence of any commercial or financial relationships that could be construed as a potential conflict of interest.

## Publisher’s note

All claims expressed in this article are solely those of the authors and do not necessarily represent those of their affiliated organizations, or those of the publisher, the editors and the reviewers. Any product that may be evaluated in this article, or claim that may be made by its manufacturer, is not guaranteed or endorsed by the publisher.
